# FACTORS ASSOCIATED WITH LOW-DOSE COMPUTED TOMOGRAPHY LUNG CANCER SCREENING IN THE UNITED STATES

**DOI:** 10.1093/geroni/igad104.2534

**Published:** 2023-12-21

**Authors:** Tung-Sung Tseng, Chien-Ching Li, Hui-Yi Lin, Yu-wen Chiu

**Affiliations:** LSUHSC, New Orleans, Louisiana, United States; Rush University, Chicago, Illinois, United States; LSUHSC, New Orleans, Louisiana, United States; LSUHSC, New Orleans, Louisiana, United States

## Abstract

Low-dose computed tomography (LDCT) screening is recommended for high-risk smokers to decrease lung cancer-related mortality. In the US, the uptake of LDCT screening among eligible smokers is suboptimal. The impacts of social-environmental and individual factors on LDCT screening uptake are not clear. The current study investigated multi-level factors associated with the utilization of LDCT screening using national data. Methods: The 2017-2021 Behavioral Risk Factor Surveillance System (BRFSS) data and other state-level variables (Medicaid expansion status and the number of screening facilities) were applied. Our study outcome is the utilization of LDCT screening among study participants who met the US Preventive Services Task Force guidelines for lung cancer screening. The final study sample consisted of 15640 respondents from 29 states. All analyses were weighted to account for the complex sampling design applied in BRFSS. Results: The overall utilization rate of LDCT screening is only 18.4%. The LDCT utilization rate varied by state (6.2 -31.1%). Among the respondents, individuals who were employed, never married, had good health status, didn’t have personal physician, experienced cost problems, never had routine checkups, and did not experience certain chronic conditions (cancer, asthma, COPD) had a lower utilization rate of LDCT screening compare with their counterpart. Conclusion: Utilization of LDCT screening among eligible smokers remains low. Risk factors associated with LDCT screening identified from this study can be used to guide future lung cancer screening programs or revise the LDCT screening guidelines to meet the needs of high-risk smokers.

